# Mutation Screening in the miR-183/96/182 Cluster in Patients With Inherited Retinal Dystrophy

**DOI:** 10.3389/fcell.2020.619641

**Published:** 2020-12-23

**Authors:** Shunbin Xu, Ardian Coku, Chithra K. Muraleedharan, Ali Harajli, Eric Mishulin, Chafic Dahabra, Joanne Choi, William J. Garcia, Kaylie Webb, David Birch, Kerry Goetz, Weifeng Li

**Affiliations:** ^1^Department of Ophthalmology, Visual and Anatomical Sciences, School of Medicine, Wayne State University, Detroit, MI, United States; ^2^Department of Biological Sciences, Wayne State University, Detroit, MI, United States; ^3^College of Literature, Science, and the Arts, University of Michigan, Ann Arbor, MI, United States; ^4^Class of 2020, School of Medicine, Wayne State University, Detroit, MI, United States; ^5^College of Natural Science, Michigan State University, East Lansing, MI, United States; ^6^Retina Foundation of the Southwest, Dallas, TX, United States; ^7^National Eye Institute, National Institutes of Health, Bethesda, MD, United States; ^8^Peking Union Medical College, Beijing, China

**Keywords:** microRNA, miR-183/96/182 cluster, inherited retinal dystrophy, sequence variants, susceptibility

## Abstract

Inherited retinal dystrophy (IRD) is a heterogenous blinding eye disease and affects more than 200,000 Americans and millions worldwide. By far, 270 protein-coding genes have been identified to cause IRD when defective. However, only one microRNA (miRNA), miR-204, has been reported to be responsible for IRD when a point-mutation occurs in its seed sequence. Previously, we identified that a conserved, polycistronic, paralogous miRNA cluster, the miR-183/96/182 cluster, is highly specifically expressed in all photoreceptors and other sensory organs; inactivation of this cluster in mice resulted in syndromic IRD with multi-sensory defects. We hypothesized that mutations in the miR-183/96/182 cluster in human cause IRD. To test this hypothesis, we perform mutation screening in the pre-miR-183, -96, -182 in >1000 peripheral blood DNA samples of patients with various forms of IRD. We identified six sequence variants, three in pre-miR-182 and three in pre-miR-96. These variants are in the pre-miRNA-182 or -96, but not in the mature miRNAs, and are unlikely to be the cause of the IRD in these patients. In spite of this, the nature and location of these sequence variants in the pre-miRNAs suggest that some may have impact on the biogenesis and maturation of miR-182 or miR-96 and potential roles in the susceptibility to diseases. Although reporting on negative results so far, our study established a system for mutation screening in the miR-183/96/182 cluster in human for a continued effort to unravel and provides deeper insight into the potential roles of miR-183/96/182 cluster in human diseases.

## Introduction

Inherited retinal dystrophy (IRD) is a heterogeneous blinding eye disease and affects more than 200,000 Americans and millions worldwide (Daiger et al., 2013). Identification of genes that are responsible for IRD when defective is of great importance to the basic understanding as well as development of efficient gene diagnosis and treatment of the disease (Xu, 2015). By far, 307 genetic loci are linked to various forms of IRD; among these, 270 protein-coding genes have been identified to cause IRD when defective (RetNet^1^. Last updated April 29, 2015). However, the roles of non-coding elements of the genome in IRDs are still not fully investigated, in spite that increasing evidences suggest that non-coding elements are critically important in the regulation of genome structures and expression of protein-coding genes, and that species-specific non-coding elements shape species-specific functions (Lander, 2011; Ulitsky and Bartel, 2013; Ransohoff et al., 2018). microRNAs (miRNAs) are endogenous, small, non-coding, regulatory RNAs (Bartel, 2004). miRNAs quantitatively regulate gene expression at posttranscriptional levels (Bartel, 2004). Since its discovery in 1993 (Lee et al., 1993; Wightman et al., 1993), miRNAs have been proven to play important roles in normal functions, as well as in diseases when defective (Ambros, 2004; Bartel, 2004). Based on miRBase Release 22.1 (October, 2018)^2^, at least 2654 mature miRNAs (1917 precursors) have been identified in humans. In spite of their importance in gene-expression regulation, by far, only one miRNA, miR-204, been identified to cause syndromic IRD with ocular coloboma when a dominant point mutation nt37 C > T in the seed sequence of miR-204 occurred in a large 5-generation family (Conte et al., 2015). The seed sequence of a miRNA is one of the most important determinants of the downstream target genes and, therefore, functions of the miRNA (Lewis et al., 2003). The nt37 C > T mutation results in both gain-of-function and loss-of-function effects, because this mutation in the seed sequence creates abnormal target genes that wild type miR-204 does not regulate, meanwhile loses control of many downstream target genes that miR-204 normally targets (Conte et al., 2015). However, no mutations in other miRNAs have been identified to cause IRD in human, although increasing evidences suggest that miRNAs are required for the normal development and functions of photoreceptors and the retina as a whole (Sanuki et al., 2011; Lumayag et al., 2013; Busskamp et al., 2014; Sundermeier et al., 2014; Aldunate et al., 2019).

Previously, we and others identified a conserved, paralogous, polycistronic miRNA cluster, the miR-183/96/182 cluster (hereafter referred to as miR-183/96/182), which is contained within 4 kilo bases (kb) on mouse chromosome 6qA3 with conservation of synteny to human chromosome 7q32.2 and is highly specifically expressed in all sensory organs (Lagos-Quintana et al., 2003; Wienholds et al., 2005; Kloosterman et al., 2006; Weston et al., 2006; Xu et al., 2007; Lumayag et al., 2013). Member of miR-183/96/182 share high sequence homology and overlapping downstream genes and, therefore, functions (Xu et al., 2007). Consistent with their high-level specific expression in sensory organs, point mutations of miR-96 result in progressive, non-syndromic hearing loss in both human (Mencia et al., 2009) and mouse (Lewis et al., 2009), with no apparent retinal phenotype, suggesting that miR-96 plays a predominant role in the inner ear, but not in retina (Lewis et al., 2009; Mencia et al., 2009; Li et al., 2010; Kuhn et al., 2011) and that members of miR-183/96/182 have distinct roles in different tissues/organs. In adult mouse retina, miR-183/96/182 is mainly expressed in all photoreceptors and a subgroup of ganglion cells. Members of miR-183/96/182 are among the highest expressed miRNAs in the retina (Xu et al., 2007; Lumayag et al., 2013). miR-182 accounts for ∼64%, miR-183 another ∼4% of all miRNAs in cone photoreceptors (Busskamp et al., 2014). Developmentally, miR-183/96/182 is minimally expressed in embryonic retina; but significantly upregulated soon after birth, suggesting that miR-183/96/182 play important roles in the postnatal functional differentiation of photoreceptors (Xu et al., 2007; Lumayag et al., 2013). Expression of miR-183/96/182 in the retina follows a diurnal rhythmic pattern – lowest around noon, highest in the early evening, suggesting a role in circadian function in the retina (Xu et al., 2007; Xu, 2009; Ko, 2020). In addition, miR-183/96/182 is responsive to light (Krol et al., 2010) – induced by light, and downregulated by darkness with 30 minutes (Krol et al., 2010). Targeted deletion of miR-182 alone in mouse did not result in a discernible phenotype, suggesting functional compensation by miR-183 and miR-96 (Jin et al., 2009). However, knockdown of miR-183/96/182 in postmitotic rod photoreceptors in a miRNA-sponge transgenic mouse model resulted in increased susceptibility to light damage in the retina (Zhu et al., 2011), with no discernible histological or functional defects in the retina under normal lighting conditions (Zhu et al., 2011), suggesting that miR-183/96/182 plays an important role in protecting the retina from light damage. We demonstrated that complete inactivation of miR-183/96/182 in mice results in syndromic IRD with multi-sensory defect (Lumayag et al., 2013), suggesting that miR-183/96/182 is required for the normal development and physiological functions of the retina and other sensory organs (Lumayag et al., 2013), providing one of the first evidences that mutation of an individual miRNA gene results in retinal dysfunction and degeneration in mammals. Our report (Lumayag et al., 2013) has been further validated by several other groups (Busskamp et al., 2014; Fan et al., 2017; Xiang et al., 2017) and our follow-up studies (Geng et al., 2018). Recent reports further substantiated critical roles of miR-183/96/182, especially miR-182/miR-183, in functional differentiation and maturation of photoreceptors (Busskamp et al., 2014; Mahmoudian-Sani et al., 2019; Peskova et al., 2020). Based on these evidences, we hypothesize that mutations in miR-183/96/182 in human also cause IRD, possibly syndromic IRD. To test this hypothesis, we established a collaboration with the National Ophthalmic Genotyping and Phenotyping Network (eyeGENE^®^)^3^, which was created by the National Eye Institute (NEI), National Institutes of Health (NIH) to enhance the study of inherited eye diseases. We obtained >1000 IRD patient DNA samples. Here we report our results in the mutation screening in these patients by far.

## Materials and Methods

### Human Genomic DNA Samples

This study was performed in accordance with the tenets of Declaration of Helsinki, and informed consent was obtained from all participants. The research was approved by the Institutional Review Board (IRB) of Wayne State University, National Institutes of Health, and Committee for Protection of Human Subjects, University of Texas Southwestern Medical Center. The eyeGENE^®^ Network constructed a framework to collect DNA samples from patients with various forms of IRD and linked them with clinical information and genetic testing data. To discover potential disease-causing mutations in miR-183/96/182 responsible for IRDs, we obtained DNA samples of IRD patients, in which no disease-causing mutations have been found in any protein-coding genes that have been screened by eyeGENE. Most testing on protein-coding genes at the eyeGENE was performed by standard commercial, fee for service panel testing, but did not go through CNV detection and evaluation of deep intronic variants.

Although the phenotypes in the miR-183/96/182 knockout mice do not completely match any human IRD, they resemble various aspects of several human IRD. The following rationales in reference to the phenotypes of the miR-183/96/182 knockout (ko) mice (Lumayag et al., 2013) were considered for sample selection: (1) since miR-183/96/182 is located at human chromosome 7q32.2, cases with X-linked IRD are excluded; (2) the most prominent ERG abnormalities in miR-183/96/182 ko mice is a decreased *b*-wave amplitude in both scotopic and photopic ERG (Lumayag et al., 2013). This phenotype resembles those observed in human incomplete Schubert-Bornschein type of stationary congenital night blindness (iSCNB) (Miyake et al., 1986); (3) the sensory syndromic feature are clinically reminiscent of Usher syndrome (Yan and Liu, 2010); (4) the cone photoreceptor system is more affected than rod photoreceptors in the ko mice; (5) it has been shown that different mutations in genes associated with IRD can cause different forms of IRD; same mutations in the orthologous genes in mouse can cause different phenotypes compared to patients (Riazuddin et al., 2010; Naeem et al., 2012; Daiger et al., 2013). Based on these considerations, we hypothesized that mutations in human miR-183/96/182 may cause one or more forms of IRD, with increased likelihood to cause SCNB, Usher syndrome and cone-rod dystrophy (CRD), and other type of cone diseases. Therefore, we first collected DNA samples of patients with SCNB (*n* = 16), Usher syndrome (*n* = 74), CRD (*n* = 248), Stargardt disease (*n* = 249), Achromatopsia/Blue Cone Monochromacy (A/BCM) (*n* = 4), Occult Macular Dystrophy (OMD. *n* = 6), Pattern Dystrophy and Adult Onset Foveomacular Dystrophy (PD and AOFD. *n* = 105) (Table 1). In addition, 509 samples with Retinitis Pigmentosa (RP) were also collected considering that miR-183/96/182 plays important roles in both rod and cones; rod system is also affected in the miR-183/96/182 ko mice. As we could not predict the inheritance pattern of a potential IRD caused by mutations in miR-183/96/182, samples with both autosomal dominant and recessive inheritance were included. DNA samples of 80 unaffected individuals were included as controls.

**TABLE 1:** DNA samples of various inherited retinal dystrophy and unaffected controls.

Disease	SCNB	Usher Syn.	Cone-rod dys.	Stargardt Disease	A/BCM	OMD	PD and AOFD	RP	Subtotal	Unaffected	Total
Number of samples	16	74	239	242	4	6	105	509	1179	80	1259

*SCNB, stationary congenital night blindness; Usher syn., Usher syndrome, Cone-rod dys., cone-rod dystrophy; A/BCM, Achromatopsia/Blue Cone Monochromacy; OMD, Occult Macular Dystrophy; PD and AOFD, Pattern Dystrophy and Adult Onset Foveomacular Dystrophy; RP, retinitis pigmentosa.*

### Amplification of Pre-miR-183/96/182 and Sanger Sequencing

**FIGURE 1:**
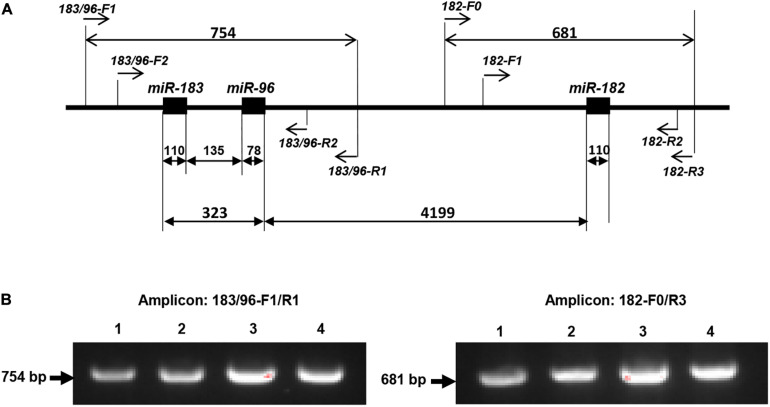
PCR-sequencing strategy for mutation screening in the miR-183/96/182 cluster. **(A)** Schematic illustration of the miR-183/96/182 cluster in relation to the PCR and sequencing primers. Pre-miR-183/96 is amplified by 183/96-F1/R1 and sequenced by primers of 183/96-F2 and 183/96-R2. pre-miR-182 was amplified with primers 182-F0/R3, and sequenced by 182-F1 and 182-R2. The numbers above the double arrows indicate the sizes of the marked fragments in base pairs (bp). **(B)** Examples of amplification products.

The miR-183/96/182 is located on human chromosome 7q32.2 and clustered with 5 kb (Xu et al., 2007). Since pre-miR-183 and pre-miR-96 are only 135 nucleotides (nt) apart, while pre-miR-182 is ∼4.2 kb 3′ of pre-miR-96 (Figure 1A), to screen miR-183/96/182, we amplified pre-miR-183, pre-miR-96 and pre-miR-182 in two amplicons (Figure 1A). pre-miR-183 and pre-miR-96 were amplified together using primers: 183/96-F1 (forward primer) and 183/96-R1 (reverse primer) (Figure 1A), which will produce a 754-bp amplification product. Pre-miR-182 was amplified separately using primers, 182-F0/182-R3 with a 681-bp amplicon.

The PCR reaction was carried out with 100 ng genomic DNA, 3 μM forward and reverse primers, 1xPCR buffer and 5 units of Taq polymerase (MIDSCI) on a S1000 thermal cycler (Bio-Rad) with the following program: 95°C, 5 min; then 95°C, 30 s; 62°C, 1 min; 72°C, 2 min for 30 cycles, followed 72°C, 10 min.

The sequences of the PCR primers are as the following:

183/96-F1: 5′-GAAGGTCATCTTGGGCTGAT-3′;

183/96-R1: 5′- CCTACAGATGGTTTCAGACTC-3′.

182-F0: 5′-TCTGGCCTGGCTTGTGCTG-3′;

182-R3: 5′-GGCTTCCCAGCTGACTTGAG-3′.

The amplification product was run on a 1.5% agarose gel to ensure the specificity (Figure 1B), before purified using the gel/PCR DNA fragment extraction kit (IBI Scientific). 50 ng of purified PCR product was sent for Sanger sequencing by the Genome Sciences Core, Wayne State University or Genewiz with the following nested sequencing primers:

For pre-miR-183/96 amplicon: 183/96-F2: 5′-GTGGATC TTGTGAAGAGGTG-3′; 183/96-R2: 5′- AGGCAGTGT AAGGCGATCTG -3′.

For pre-miR-182 amplicon: 182-F1: 5′-ACAGGAACT GCAGGTTACAGA -3′; 182-R2: 5′- CTTGAGGACCTGT GACCTCA-3′.

Sequences obtained from sequencing using the forward primers (183/96-F2 or 182-F1) were aligned with the reference sequence downloaded from the UCSC Genome Browser [Human Dec. 2013 (GRCh38/hg38) Assembly] (Supplementary Material) using Vector NTI 11.0 (Invitrogen). All sequence variants identified by the sequence alignment were confirmed by the sequencing using the reverse primers (183/96-R2 or 182-R2).

### Copy Number Assay

Patient genomic DNA was extracted from blood using the IBI gMAX mini genomic kit. DNA was diluted to 5 ng/μL with nuclease–free water. 4 μl of diluted DNA was added per well in a 96 well plate. Custom designed TaqMan^®^ Copy Number Assay (4400294. Assay ID: MIR182_CDYMJXM) for pre-miR-182 was added along with TaqMan^®^ Copy Number Reference Assay *RNase P*. The reactions were run on an Applied Biosystems 7500 Fast Real-Time PCR System using the following cycle parameters: 95°C, 10 min; then 95°C, 15 s; 60°C, 60 s for 40 cycles. Data generated was analyzed using Applied Biosystems CopyCaller^®^ Software v2.0.

### Isolation of Mononuclear Cells From Peripheral Blood

Peripheral blood mononuclear cells (PBMNC) were isolated from human peripheral blood (with EDTA or heparin as anti-coagulant) using the Histopaque-1077 (Sigma-Aldrich. Cat No. 10771). Manufacturer’s instruction was followed. Briefly, 3 ml of peripheral blood was carefully layered on 3 ml Histopaque-1077 in a 15-ml conical tube and centrifuged at 400 × *g* for 30 min at room temperature. After centrifugation, the upper plasma layer was aspirated off. Then the PBMNC layer was carefully removed and washed in cold PBS. The cells are divided into two tubes for total RNA isolation using the RNeasy RNA isolation kit (Qiagen) and miRNA isolation using the miRVana miRNA isolation kit (Ambion). RNA concentration and quality were analyzed using a NanoDrop 2000 (Thermo Fisher Scientific).

### Genomic DNA Preparation

Two milliliters of peripheral blood was used for genomic DNA preparation using the gMAX DNA mini kit (IBI Scientific) following manufacturer’s instruction. Subsequently, DNA concentration and quality were assayed on the NanoDrop 2000 (Thermo Fisher Scientific).

### Quantitative (q)RT-PCR of miR-182

qRT-PCR was performed using TaqMan miRNA primers and RT-PCR kit (Life Technologies) on a CFX Connect Real-time System (Bio-Rad, Hercules, CA, United States) with snRNA U6 as an endogenous control as described before.

### Secondary Structure Prediction of Hsa-pre-miR-182 and Hsa-pre-miR-96

The sequences of hsa-pre-miR-182 and hsa-pre-miR-96 were downloaded from miRBase^4^. The residues of sequence variants identified in this study were modified to obtain the sequences of the mutant forms of hsa-pre-miR-182 and hsa-pre-miR-96. Then these sequences were uploaded to the RNAfold web server^5^ to obtain predicted secondary structures with minimum free energy (MFE) and partition function (Mathews et al., 2004; Gruber et al., 2008; Lorenz et al., 2011).

### Statistical Analysis

When the comparison was made among more than two conditions, One-way ANOVA with Bonferroni’s multiple comparison test was employed (GraphPad Prism); adjusted *p* < 0.05 was considered significant. Otherwise, a two-tailed Student’s *t* test was used to determine the significance; *p* < 0.05 was considered significant. The significance of the association of sequence variants to various disease categories in this cohort was compared to general population by Fisher’s exact test, or Chi square test for the tri-allelic SNP rs80041074 (pre-miR-182 nt94 G > A).

## Results

**FIGURE 2:**
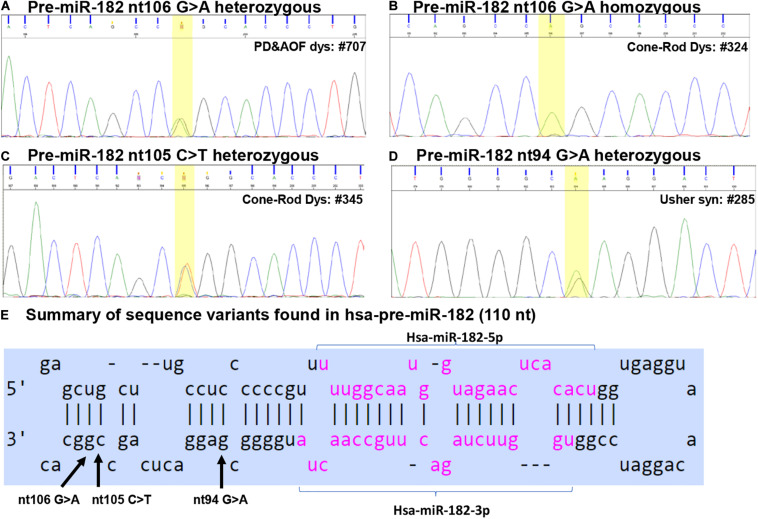
Sequence variants identified in pre-miR-182. **(A–D)** Example of Sanger sequencing chromatography of patient DNA samples which are heterozygous for pre-miR-182 nt106 G/A alleles **(A)** and homozygous for pre-miR-182 nt106 A/A allele **(B)**, heterozygous for pre-miR-182 nt105 C > T **(C)** and nt94 G > A variants **(D)**. **(E)** Summary of the sequence variants of pre-miR-182 in the context of the hairpin structures of hsa-miR-182. Reference: miRbase.org.

**FIGURE 3:**
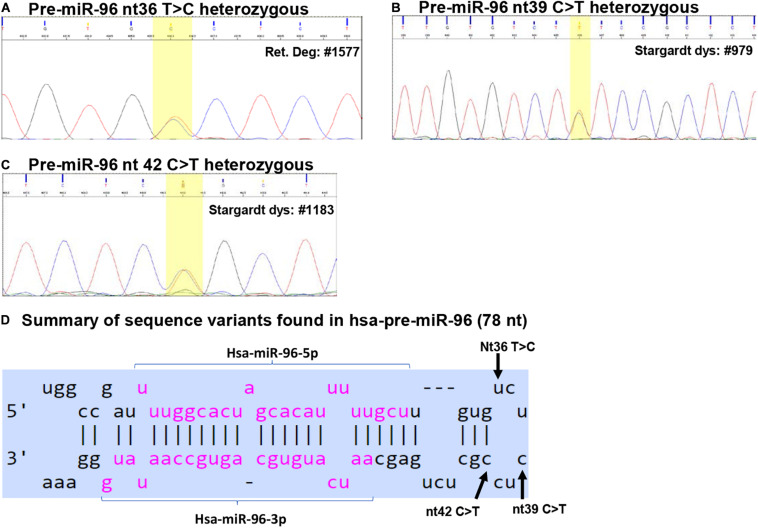
Sequence variants identified in pre-miR-96. **(A–C)** Example of Sanger sequencing chromatography of patient DNA samples which are heterozygous for pre-miR-96 nt36 T > C **(A)**, nt39 C > T **(B)**, and nt42 C > T variants **(C)**. **(D)** Summary of the sequence variants of pre-miR-96 in the context of the hairpin structures of pre-miR-96. Reference: miRbase.org.

Among the 1179 IRD patient samples (Table 1), we identified three sequence variants in the pre-miR-182: nt106 G > A, nt105 C > T and nt94 G > A (Figure 2), and three sequence variants in pre-miR-94: nt36 T > C, nt39 C > T and nt42 C > T (Figure 3).

### Sequence Variants in Hsa-pre-miR-182

#### Pre-miR-182 nt106 G > A

This variant was found in 32 IRD patients, including two in CSNB patients, nine CRD, one Usher syndrome, four PD and AOFD, three Stargardt disease patients, and 13 patients with RP (Table 2 and Figures 2A,B). Among these, we found homozygosity for the less common A allele in five patients including one CRD, two Stargardt, and two RP patients (Table 2). Copy-number assay confirmed that these cases are true homozygosity for this variant instead of a micro-deletion in one allele. In 80 unaffected individuals, we identified one individual heterozygous for pre-miR-182 nt106 G/A. The A allele frequency in our samples is as the following: overall frequency in IRD patients: 1.569%; in CSNB patients: 6.250%; Usher syndrome: 0.676%; CRD: 2.092%; Stargardt dis.: 1.033%; PD and AOFD: 1.905%; other RP: 1.473% (Table 2). In unaffected individual: 0.625%.

**TABLE 2:** Allele frequency of the sequence variants among the samples.

Disease	Variant	nt106 G > A			nt105 C > T		nt94 G > A		nt36 T > C		nt39 C > T		nt42 C > T	
		pre-miR-182			pre-miR-182		pre-miR-182		pre-miR-96		pre-miR-96		pre-miR-96	
		(rs76481776)			(rs77586312)		(rs80041074)		(rs41274239)		(rs73159662)		(rs73159662)	
	Freq in dbSNP	G: 94.048%; A: 5.952%			C: 99.848%; T: 0.152%		G: 99.61%; A: 0.389%; T: 0.001%		T: 99.752%; C: 0.249%		C:99.99%; T: 0.01%		C:99.713%; T: 0.287%	
							
	No	No	No of homo	freq (%)	No	freq (%)	No	freq (%)	No	freq (%)	No	freq (%)	No	freq (%)
SCNB	16	2		6.250	1	3.125*								
Usher syn.	74	1		0.676**			1	0.676						
Cone-rod dys.	239	9	1	2.092***	1	0.209								
Stargardt disease	242	3	2	1.033***			2	0.413			1	0.207	2	0.413
A/BCM	4													
OMD	6													
PD and AOFD	105	4		1.905**			1	0.476						
Other RP	509	13	2	1.473***			3	0.295	3	0.295				
Subtotal	1179	32	5	1.569***	2	0.085	7	0.297	3	0.127	1	0.042	2	0.085
Un-affected	80	1		0.625			1	0.625						
Total	1261	65		1.507	4	0.079	15	0.317	6	0.119	2	0.04	4	0.079

Freq, frequency; No, number; homo, homozygote; SCNB, stationary congenital night blindness; Usher syn., Usher syndrome; Cone-rod dys., cone-rod dystrophy; A/BCM, Achromatopsia/Blue Cone Monochromacy; OMD, Occult Macular Dystrophy; PD and AOFD, Pattern Dystrophy and Adult Onset Foveomacular Dystrophy; RP, retinitis pigmentosa. *, **, ***: *p* < 0.05, 0.01, and 0.001 when compared to general population data compiled by dbSNP by Fisher’s exact test.

The pre-miR-182 nt106 G > A variant is a known single nucleotide polymorphism (SNP) documented in the dbSNP as rs76481776 with the allele frequencies of 5.952% (7211/121160) for the A allele, while 94.048% (113949/121160) for the G allele based on data from UCSC genome browser (dbSNP build 151). Statistical analysis suggest that the pre-miR-182 A allele appears to be significantly lower in all groups of patients and overall IRD population in this cohort, except in CSNB patients (Table 2).

**FIGURE 4:**
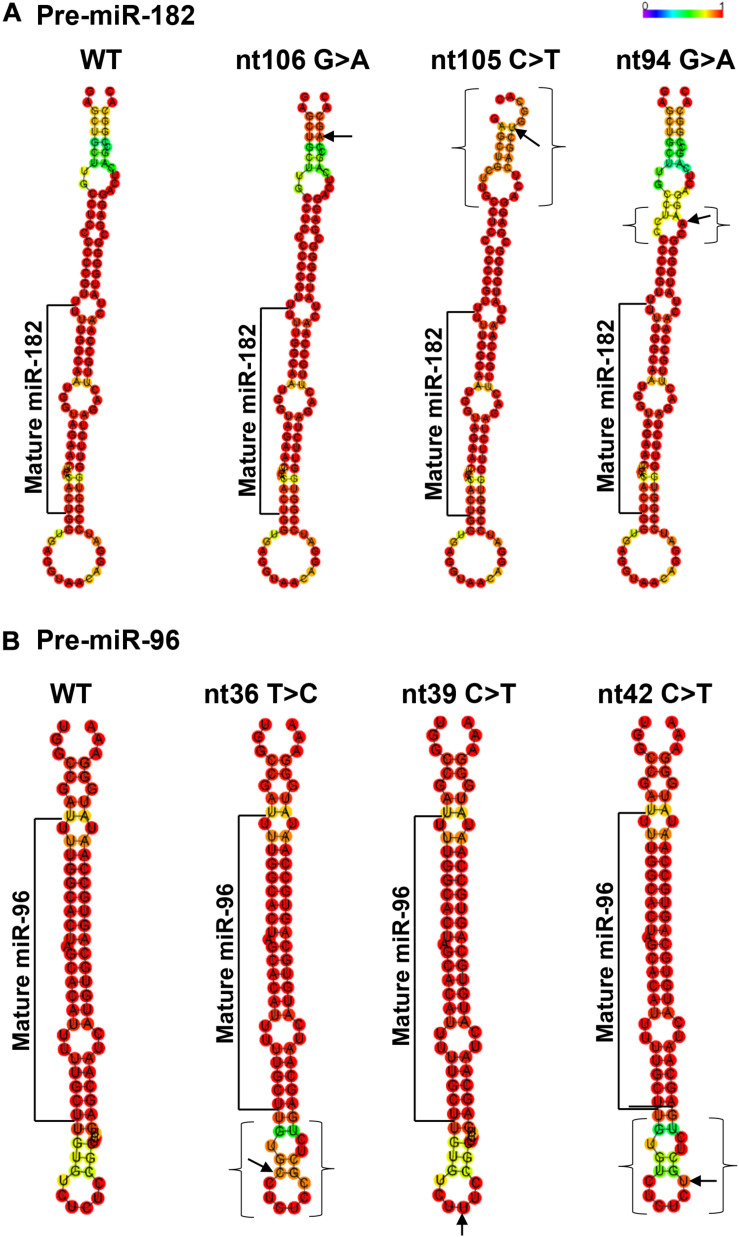
Minimum free energy secondary structure prediction with base-pair probability on wild type (WT) hsa-pre-miR-182 **(A)** and hsa-pre-miR-96 **(B)** and their mutant forms with sequence variants. Data was compiled using publicly available software *RNAfold* (http://rna.tbi. univie.ac.at//cgi-bin/RNAWebSuite/RNAfold.cgi). Arrows indicate the sequence variant nucleotide. The brackets mark the areas with predicted changes of the secondary structure because of the sequence variants.

The pre-miR-182 nt106 G > A is located at the 5th nucleotide from the 3′ end of the hairpin structure of the human pre-miR-182 (Figure 2E). It is predicted that nt106 G > A change results in a transition of G•U wobble base-pairing to a classical Watson-Crick base pair A•U, suggesting potential functional significance (Varani and McClain, 2000). However, a secondary structure prediction using the RNAfold algorithm (Mathews et al., 2004; Gruber et al., 2008; Lorenz et al., 2011)^6^ showed little changes in the secondary structure and the minimum free energy of the hair-pin structure of pre-miR-182 (Figure 4A and Table 3).

**TABLE 3:** Changes in the predicted secondary structures and their free energy caused by the variants of pre-miR-182 (A) and pre-miR-96 (B).

Pre-miR-182	WT	nt106 G > A	nt94 G > A	nt105 C > T
Minimum free energy (MFE)	–47.10	–47.80	–43.80	–48.60
Free energy of the thermodynamic ensemble	–48.41	–49.03	–45.26	–49.19
Frequency of the MFE structure (%)	11.89	13.55	9.41	38.11
Ensemble diversity	7.40	6.27	9.30	4.64

**Pre-miR-96**	**WT**	**nt36 T > C**	**nt39 C > T**	**nt42 C > T**

Minimum free energy (MFE)	–34.80	–35.60	34.80	–33.60
Free energy of the thermodynamic ensemble	–35.26	–36.19	35.26	–34.37
Frequency of the MFE structure	47.49	38.16	47.51	28.84
Ensemble diversity	2.90	2.42	2.90	2.97

#### Pre-miR-182 nt105 C > T

Pre-miR-182 nt105 C > T was identified in one CSNB and one CRD patient (Table 2 and Figure 2C), which are heterozygous for this allele. The overall frequency of the T allele in our IRD cohort: 0.085%. In SCNB: 3.125%; in CRD: 0.209% (Table 2). This variant was not identified in any unaffected individuals. This variant is documented in dbSNP as a rare SNP rs77586312: with frequencies of the T allele 0.152% (184/121378), while the C allele 99.848% (121194/121378). Comparing to the general population data compiled at dbSNP, the rare pre-miR-183 nt105 T appears to be significantly enriched in CSNB patients in this cohort.

This variant is also near the 3′ end of the hsa-pre-miR-182, located at the 6th nucleotide from the 3′ end (Figure 2E). It results in a change of the classical Watson-Crick base pair G•C to a G•U wobble base pair. Secondary structure prediction by RNAfold suggest that nt105 C > T variant may induce changes in the basal segment of the stem of pre-miR-182 (the bracketed area in Figure 4A), although it did not cause drastic changes of the minimum free energy (Table 3).

#### Pre-miR-182 nt94 G > A

Pre-miR-182 nt94 G > A was identified in one Usher syndrome, two unrelated Stargardt disease, one PD and AOFD and three unrelated other RP patients, as well as in one unaffected individual (Table 2 and Figure 2D). All are heterozygous for this allele. The overall frequency of this variant in all IRD patients in this cohort was 0.297%. It has a frequency of 0.676% in Usher syndrome; 0.413% in Stargardt disease; 0.476% in PD and AOFD; 0.295% in other RP patients. In unaffected individuals: 0.625% (Table 2).

Pre-miR-182 t94 G > A is documented as a rare tri-allelic SNP rs80041074 with minor allele frequency as A 0.389% (475/121998), T allele 0.001% (1/121998), while the common allele G has a frequency of 99.61% (121522/121998) in the dbSNP (dbSNP build 151).

This SNP is located at the 17th nucleotide from the 3′ end of the hsa-pre-miR-182. It disrupts a C•G in the predicted hair-pin structure (Figure 2E). Secondary structure prediction by RNAfold showed that nt94 G > A change may induce changes in the size of the bulge (bracketed area in Figure 4A) of the secondary structure of pre-miR-182, resulting in an increase of the minimum free energy level (Table 3. from −47.10 to −43.80) at its thermodynamic ground state. However, whether these changes impose any impact on miR-182 maturation need to be test experimentally.

### Sequence Variants in Hsa-pre-miR-96

We identified three sequence variants in pre-miR-94: nt36 T > C, nt39 C > T and nt42 C > T (Figure 3).

#### Pre-miR-96 nt36 T > C

The pre-miR-96 nt36 T > C was identified in three unrelated RP patients heterozygous for this allele, but not in any other IRD patients and unaffected controls (Table 2 and Figure 3A). The minor C allele has a frequency of 0.295% in RP patients, 0.127% in all IRD patients. This variant is located in the loop region of hsa-pre-miR-96 (Figure 3D).

The pre-miR-96 nt36 T > C variant is known as a rare SNP rs41274239 with allele frequencies of 0.249% (65/26162) for the minor C allele; 99.752% (26097/26162) for the common T allele based on data from UCSC genome browser (dbSNP build 151).

Secondary structure prediction using RNAfold showed that pre-miR-96 nt36 T > C may change the landscape of the terminal loop of pre-miR-96 (the bracketed area in Figure 4B) at its thermodynamic ground state, although this change did not induce drastic changes in the free energy of the secondary structure of pre-miR-96 (Table 3).

#### Pre-miR-96 nt39 C > T

The pre-miR-96 nt39 C > T variant was identified in one Stargardt disease patient who is heterozygous for this allele (Table 2 and Figure 3B). In the study cohort, the minor T allele has a frequency of 0.207% in Stargardt disease patients, and 0.042% in all IRD patients. None was detected in the unaffected samples (Table 2). This variant is documented as rare SNP rs73159662: C: 99.990% (10181/10182); T: 0.010% (1/10182) in dbSNP (build 151).

This variant is also located in the stem loop region of the hsa-miR-96 (Figure 3D). Secondary structure prediction by RNAfold program did not expect significant changes in the thermodynamic status of the secondary structure of pre-miR-96 (Figure 4B and Table 3).

#### Pre-miR-96 nt42 C > T

This variant was identified in two unrelated Stargardt disease patients heterozygous for this allele. In the study cohort, the minor T allele in Stargardt disease patient is 0.413%, while the overall frequency in all IRD is 0.085% (Table 2).

Pre-miR-96 nt42 C > T variant is documented as a rare SNP rs73159662 with the minor allele T frequency as: 0.287% (75/26094), the common C allele 99.713% (26019/26095) in the dbSNP (build 151).

This variant is predicted to disrupt the classical Watson-Crick base pair G•C at the first residue after the terminal loop of the predicted hsa-pre-miR-96 structure (miRbase) (Figure 3D), suggesting potential impact on the structures around the terminal loop. Secondary structure prediction by RNAfold algorithm suggested that nt42 C > T may induce similar changes in the terminal loop of pre-miR-96 at its thermodynamic ground state, as did the pre-miR-96 nt36 T > C variant (the bracketed area in Figure 4B), although it did not induce drastic changes in the free energy of the secondary structure of pre-miR-96 (Table 3). Since the terminal loops of pre-miRNAs can play important roles in miRNA biogenesis (Castilla-Llorente et al., 2013; Treiber et al., 2019), whether these variants impose significant impact on miR-96 maturation should be further studied.

#### Segregation Study in a Family, Family 4100, of a Proband Homozygous for Pre-miR-182 nt106 G > A Variant

In spite that the pre-miR-182 nt106 G > A is common variant with a frequency of 5.952% in general population (dbSNP), it was reported that minor A allele is associated with higher risk of late insomnia in major depression patients (Saus et al., 2010). *In vitro* expression studies showed that the A allele resulted in an increased production of mature miR-182 and enhanced inhibition of its target genes (Saus et al., 2010). In addition, pre-miR-182 nt106 G > A is also reported to be associated with high tension glaucoma (HTG), but not normal tension glaucoma (NTG); the A allele gives 30% higher expression of mature miR-182 *in vitro* transfection/expression experiment (Liu et al., 2016). Furthermore, this variant is also shown to be associated with genetic susceptibility to Behcet’s disease and Vogt-Koyanagi-Harada (VKH) syndrome, both of which have autoimmune uveitis; mature miR-182 showed an increased expression level in activated CD4^+^ T cells of the patients with nt106 A allele than normal controls (Yu et al., 2014). These reports suggest that pre-miR-182 nt106 G > A variant may have significant impact on the maturation of miR-182, which play important roles in the differentiation of photoreceptors and the function of the retina and other sensory systems (Lumayag et al., 2013; Busskamp et al., 2014; Fan et al., 2017; Mahmoudian-Sani et al., 2019; Peskova et al., 2020). Consistent with this report, recent reports from us and others have shown that miR-183/96/182, including miR-182, is also expressed and plays important roles in the immune systems and autoimmune diseases (Stittrich et al., 2010; Donatelli et al., 2014; Ichiyama et al., 2016; Li et al., 2016; Muraleedharan et al., 2016, 2019; Wan et al., 2016; Wurm et al., 2017; Wang et al., 2019).

**FIGURE 5:**
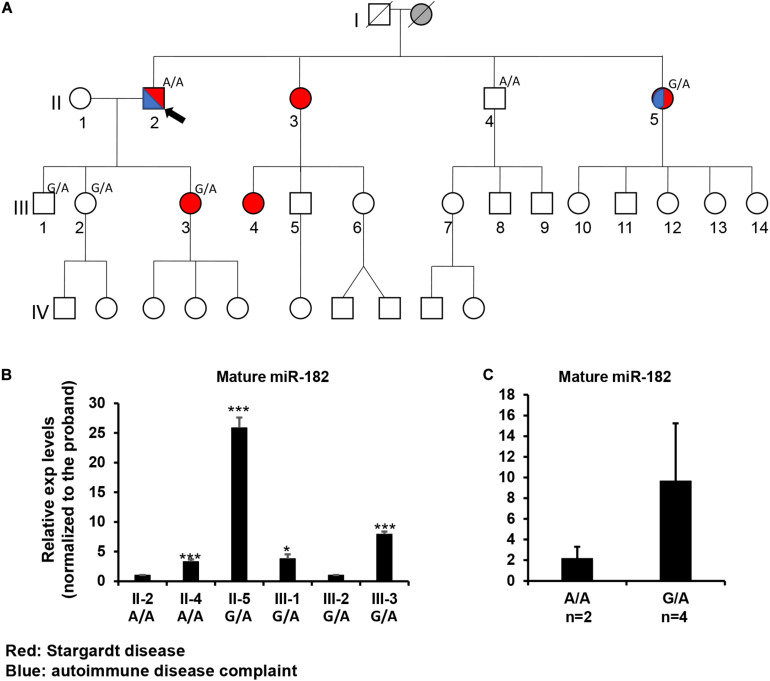
Inheritance patterns of the pre-miR-182 nt106 G/A allele and the expression levels of mature miR-182 in peripheral blood mononuclear cells (PBMNC) in a family with multiple cases of Stargardt disease -Family 4100. **(A)** the family pedigree. Proband II-2 and his brother II-4 are homozygous for the less common pre-miR-182 nt106 A allele (A/A); while II-5, III-1, 2, 3 are heterozygous (G/A) for this allele. Red: with Stargardt disease. Blue: with Complaint of autoimmune disease(s). **(B,C)** qRT-PCR on pre-miR-182 in the PBMNC of selected family members. *p < 0.05; ***p < 0.001.

If the A allele does affect the miR-182 maturation and contribute to various diseases, we hypothesized that homozygosity of pre-miR-182 nt106 G > A may have significant functional impact on mature miR-182 expression and contribute to syndromic IRD. To test this hypothesis, we collected DNA samples from several members of a family – Family 4100, in which multiple members have Stargardt disease and the proband, II-2, is homozygous for the less common, pre-miR-182 nt106 A allele (A/A) (Figure 5A). To test whether the Stargardt disease is associated with syndromic immune defects, we expanded our clinical observation to include both retinal and immune/inflammatory symptoms. The proband was diagnosed to have Stargardt disease at age of 65 in 2019 with clear vitreous, normal optic nerve, normal retinal vessels, pigmentary changes throughout macula, GA OU (both eyes), mfERG results in 2013 were abnormal surrounding the fovea, visual fields showed central scotoma and general reduction of sensitivity. However, his vision acuity has stayed remarkably good (20/20 and 20/50 in 2019). Patient reports unknown autoimmune ear disease leading to a sudden and permanent hearing loss in the left ear in 2004, and thought to be attributed to psoriatic arthritis. Patient also reports mild flares of arthritis in thumbs and knee. One sister of the proband, II-3, was diagnosed with Stargardt disease in 2016 with pigmentary changes throughout macula (flecks). She has no reported auto-immune disease diagnosed. Another sister of the proband, II-5, is also diagnosed with Stargardt disease and was reported to have Lupus-like symptoms, although they have not been accurately diagnosed.

To test whether the Stargardt disease and/or the autoimmune symptoms segregate with the A/A homozygosity, blood samples were collected from individual II2, 4, 5 and III-1,2,3. Unfortunately, genotyping on the pre-miR-182 showed that neither the Stargardt disease nor the autoimmune symptoms segregated with pre-miR-182 nt106 A/A homozygosity (Figure 5A). II-4, homozygous to A/A, is phenotypically normal; while II-5 and III-3, who are heterozygous (G/A) for the pre-miR-182 nt106 allele, are affected with Stargardt disease, as well as autoimmune symptom (for II-5) (Figure 5A). Therefore, homozygosity of pre-miR-182 nt106 A/A is unlikely to be the cause of the Stargardt disease and autoimmune symptoms in this family.

To test whether the pre-miR-182 nt106 G > A variant has any impact on the expression levels of mature miR-182, we isolated RNA from PBMNC in these family members. qRT-PCR result showed no clear correlation between the expression levels of mature miR-182 and the genotype of pre-miR-182 nt106 alleles (A/A or G/A) (Figures 5B,C). Homozygosity for the pre-miR-182 nt106 A allele (A/A) did not show increased expression of mature miR-182, as described by previous reports (Saus et al., 2010; Yu et al., 2014; Liu et al., 2016). On the contrary, mature miR-182 in PBMNC from individuals with G/A allele had an increasing trend than the ones with A/A allele (Figures 5B,C), although the difference did not pass statistical significance threshold, because of limited number of samples.

## Discussion

By far, only three miRNAs, miR-96, miR-184 and miR-204, have been identified to cause inherited diseases in human when mutated. Point mutations in the seed region of the mature miR-96 (nt13 G > A and nt14 C > A) cause autosomal dominant non-syndromic hearing loss (Mencia et al., 2009). A point mutation in the seed sequence of miR-184 (nt57 C > U) is reported to cause autosomal dominant, familial keratoconus with early onset anterior polar cataract (Hughes et al., 2011) and an autosomal dominant syndromic anterior segment dysgenesis – the endothelial dystrophy, iris hypoplasia, congenital cataract, and stromal thinning (EDICT) syndrome (Iliff et al., 2012). A dominant mutation in miR-204, nt37 C > T, causes an autosomal dominant IRD associated with bilateral ocular coloboma (Conte et al., 2015) – the first and only report that a point mutation in an individual miRNA results in significant functional consequence and causes IRD in human. In these three cases, the disease-causing mutations are all in the seed sequences of mature miRNAs. The seed sequence of a miRNA is one of the most important determinants of its downstream target genes and hence functions (Lewis et al., 2003). These mutations in the seed sequences of miR-96, miR-184, and miR-204 resulted in both loss and gain of function effects – loss of control of normal target genes while creating new, abnormal target genes, leading to global dysregulation of gene expression and dominant inherited diseases (Mencia et al., 2009; Hughes et al., 2011; Iliff et al., 2012; Conte et al., 2015). Here we identified three sequence variants in pre-miR-182; three sequence variants in pre-miR-96 among 1179 patients with various forms of IRD. All of these sequence variants are located in the pre-miR-182 or pre-miR-96, but not in the mature miR-182 or miR-96. Therefore, they won’t have any impact on the spectrum of downstream targets of miR-182 or miR-96. In addition, these variants are all known SNPs which have been identified in the general population and reported in the dbSNP. Therefore, the sequence variants that we identified so far are less likely to be the disease-causing mutations responsible for the IRD symptoms in these patients. In the CSNB group, the rare pre-miR-182 nt105 T appears to be significantly enriched in CSNB patients when compared to the general population data compiled at dbSNP; however, we only have a small number of CSNB patients in this cohort; whether this “enrichment” is of biological significance or a random bias because of the small number of samples needs to be further investigated in future studies with more CSNB patient samples. In our cohort of a total of 1179 patients, five probands (0.42%) were found homozygous for the pre-miR-182 nt106 G > A (rs76481776). Although there is no data on the homozygosity of this variant in the general population, it is reasonable to predict a ∼0.35% frequency in the general population based on its 5.952% allele frequency, which is similar to the frequency in our cohort (0.42%). In Family 4100, in which multiple members are diagnosed with Stargardt disease and the proband is homozygous for the minor pre-miR-182 nt106 A allele, homozygosity of the A allele did not segregate with either the Stargardt disease or autoimmune symptoms in members of this family, suggesting that it is unlikely to be the cause for these diseases. While we were investigating on mutations in miR-183/96/182, eyeGENE continued on the screening in protein-coding genes and identified that the proband of Family 4100 was heterozygous for ABCA4 c.5461-10T > C VUS (variant of uncertain significance). This variant is located in intron 38 of the ABCA4 gene. It was first discovered in 1999 in Stargardt disease patients (Maugeri et al., 1999), and has been described as the third most frequent gene variant associated with Stargardt disease in individuals with European or African descent (Zernant et al., 2011; Roberts et al., 2012; Miraldi Utz et al., 2014). Although it was initially identified as a sequence variant without significant functional consequence (Rivera et al., 2000), recent reports suggest that this mutation results in a decreased expression level of wild type ABCA4 and splicing defects with exons 39/40 skipped, leading to frameshift and premature stop, playing a causative and a pathological role in Stargardt disease (Sangermano et al., 2016; Aukrust et al., 2017; Jonsson et al., 2018). We predict that the intronic mutation, c.5461-10T > C in ABCA4, contributes to the Stargardt disease in Family 4100. Further investigation is required to determine whether the disease in this family is solely caused by the ABCA4 c.5461-10T > C variant or whether mutations in other genes are involved.

Therefore, we haven’t yet identified disease-causing mutations in the miR-183/96/182 cluster in human. However, this is the first stage of a long-term study. The negative results so far cannot exclude miR-183/96/182 as a disease-causing gene in these IRD patients yet. In current study, the mutation screening is purely focused on the sequences of pre-miR-183/-96/-182. In the next stage of our study, we plan to sequence the entire genomic region of the miR-183/96/182 gene to test whether there are mutations in the promoter/enhancer and other regions of the miR-183/96/182 cluster gene which may affect the transcription and post-transcriptional regulation of the miR-183/96/182 cluster and contribute to or cause IRD in these patients. We are aware of the challenges to the identification of disease-causing mutations in this cohort. First, most IRD patients are sporadic cases without family history, inheritance pattern and DNA samples from other members of the family. Second, no RNA and other tissue samples from the same patients are available to test the expression levels of miR-183/96/182 in the retina or other tissues. To meet these challenges, as we showed in Family 4100, we extended our collaboration with dedicated physician scientists and candidate family members to obtain additional family history and samples to make conclusive studies, once clues are identified in the initial screening. In addition, future studies including Crispr-Cas9-mediated mutagenesis in iPS cells in combination of *in vitro* organogenesis will further help meet these challenges.

**FIGURE 6:**
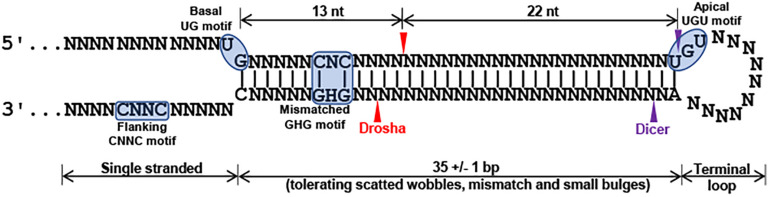
Structural and primary sequence features of pri-miRNA stem of canonical miRNA hairpin (modified from Fang and Bartel, 2015; Nguyen et al., 2015; Bartel, 2018). A canonical miRNA hairpin includes a 35 +/− 1 bp stem, an unstructured terminal loop and singled stranded flanking regions. A basal UG motif, a mismatched GHG motif (H = A, C or U), an apical UGU motif, and flanking CNNC (N = any of the four nucleotides) motif enhance the processing and the precision of Drosha cleavage (red arrowheads). Purple arrowheads mark the sites for DICER cleavage.

In spite that we have not yet identified disease-causing mutations, studies on the rare sequence variants have stimulated greater insights into the potential of mutations of miR-183/96/182 in human diseases. Although these sequence variants are not expected to change the spectrum of downstream target genes of miR-182 or miR-96, we do not exclude the possibility that they have significant impact on miRNA biogenesis and maturation of the miR-182 or miR-96. miRNA biogenesis involves two RNase III-type enzymes, DROSHA in the nucleus (Lee et al., 2003; Denli et al., 2004; Han et al., 2004; Landthaler et al., 2004) and DICER in the cytosol (Grishok et al., 2001; Hutvagner et al., 2001). Drosha and a dimer of its cofactor DGCR8 form the core of a heterotrimeric Microprocessor (Nguyen et al., 2015), which essentially determines the mature miRNA sequence, as Drosha generates the termini of pre-miRNA, from which ∼22 nt are measured by DICER (Auyeung et al., 2013; Fang and Bartel, 2015; Kim et al., 2017). The mammalian Microprocessor prefers a stem of 35 ± 1 bp, a terminal unstructured loop and single-stranded regions flanking the base of hairpin (Figure 6; Fang and Bartel, 2015; Bartel, 2018). Throughout the stem, pairing is preferred, although a few mismatches or small bulges are tolerated. Several features of the hairpin structure of a pre-miRNA enhances the processing and help specify the sites of cleavage by Drosha (Figure 6; Auyeung et al., 2013; Fang and Bartel, 2015; Bartel, 2018). Drosha recognizes the base of the hairpin, including the basal UG motif and the mismatched GHG motif (H = A, C or U), while DGCR8 dimer recognizes the apical region, including the apical UGU motif. Drosha and DGCR8 dimer act as a molecular caliper to measure the length of the stem (Auyeung et al., 2013; Fang and Bartel, 2015; Nguyen et al., 2015; Bartel, 2018; Figure 6). A 3′ flanking CNNC motif, ∼17–18 nt downstream of the Drosha cleavage site (Auyeung et al., 2013), is recognized by auxiliary factors, e.g., SRp20 or p72, to enhance the processing (Auyeung et al., 2013; Mori et al., 2014; Bartel, 2018; Figure 6). The pre-miR-182 nt94 G > A variant is located in the vicinity of the GHG motif; while pre-miR-182 nt106 G > A and nt105 C > T are located in the CNNC motif of the pri-miR-182 hairpin structure. These sequence variants may modulate the processing by the Microprocessor and result in changes in the production of mature miR-182. Our preliminary study on the expression of mature miR-182 in PBMNCs of the family with Stargardt disease (Figure 5C) showed a trend consistent with this hypothesis. The average expression levels of mature miR-182 in the individuals with homozygous nt106 A/A allele showed a decreased trend when compared to the ones heterozygous for this allele (nt106 G/A). However, we only obtained two samples with homozygous nt106 A/A allele because of its rarity, therefore, cannot make statistically significant conclusion yet. Further larger-scale study is needed to validate this result and prove our hypothesis. In addition, we’d clarify that the current study serves as the discovery stage of a long-term project. We will perform downstream experiments to evaluate the functional consequences and potential roles in diseases of all other variants in the future.

Intriguingly, all three sequence variants identified in pre-miR-96 are in the terminal loop of pri-miR-96 hairpin structure. Recent reports demonstrate that increasing number of RNA binding proteins (RBPs) recognize the terminal loops of selective pri-miRNA hairpin structures, e.g., Lin28a (Viswanathan et al., 2008; Nam et al., 2011), hnRNPA1 (Guil and Caceres, 2007; Michlewski et al., 2008; Kooshapur et al., 2018), KSRP (Trabucchi et al., 2009; Michlewski and Caceres, 2010), to modulate the processing of pri-miRNAs by the Microprocessor and/or pre-miRNA by Dicer (Castilla-Llorente et al., 2013; Treiber et al., 2019). The temporal and tissue specificity of the expression of these RBPs may play a role in defining the expression pattern and function of selective miRNAs at a given time and in a given cell type (Castilla-Llorente et al., 2013). It is reasonable to speculate that the sequence variants in pre-miR-96 may have consequential impact on the maturation and expression levels of mature miR-96. Since miR-182 and miR-96 are among the highest expressed miRNAs and play important roles in the functional differentiation of photoreceptors, hair cells and other sensory neurons (Weston et al., 2006, 2011; Xu et al., 2007; Lumayag et al., 2013; Busskamp et al., 2014; Fan et al., 2017; Geng et al., 2018), if these sequence variants have significant influence on the production of mature miR-182 or miR-96, we further hypothesize that these changes could impose quantitative modulation on the functions of photoreceptors and other sensory neurons and confer susceptibility to various types of IRDs and syndromic diseases in other sensory organs.

As we mentioned earlier, recent discoveries from us and other groups have greatly expanded our knowledge on the miR-183/96/182 cluster. In addition to the sensory organs, miR-183/96/182 cluster plays important roles in both innate and adaptive immune systems. In innate immune system, we demonstrated that miR-183/96/182 cluster is expressed in macrophages and neutrophils and modulates their phagocytosis and intracellular bacterial killing capacity and their inflammatory response to bacterial infection through targeting Nox2 and DAP12 (Muraleedharan et al., 2016, 2019). Inactivation of miR-183/96/182 cluster in mice resulted in increased phagocytosis and bactericidal capacity and decreased production of pro-inflammatory cytokines in response to *Pseudomonas aeruginosa* (PA) infection and decreased overall severity of PA keratitis (Muraleedharan et al., 2016, 2019). Wurm et al. further discovered that miR-182 modulates granulocytic differentiation through a negative regulatory loop with targeting transcription factor C/EBPα (Wurm et al., 2017). C/EBPα is a master regulator of myelopoiesis. It blocks miR-182 expression by direct promoter binding during myeloid differentiation; while miR-182 targets C/EBPα and impairs myelopoiesis and granulocytic differentiation (Wurm et al., 2017). miR-182 expression is highly elevated in acute myeloid leukemia (AML) patients with C/EBPα mutations disrupting its C-terminal DNA binding domain and is recommended as a prognostic marker in cytogenetically high-risk AML patients (Wurm et al., 2017). In natural killer (NK) cells, miR-183/96/182 cluster is induced by TGFβ and mediate TGFβ-induced inhibition of NK cell functions, e.g., tumor cytolysis, through targeting DAP12 (Donatelli et al., 2014).

In adaptive immune system, we showed that miR-183/96/182 is involved in invariant NKT cell development, maturation and effector functions (Wang et al., 2019). miR-182 and miR-183, but not miR-96, are shown to be drastically induced in activated helper T (Th) cells through the IL-2/CD25/STAT5 pathway (Stittrich et al., 2010). miR-182 promotes clonal expansion of activated Th cells through targeting Foxo1 (Stittrich et al., 2010). Furthermore, we and our collaborators further showed that in Th17 cells, induced by IL-6-STAT3 signaling, the miR-183/96/182 cluster promotes Th17 pathogenicity by targeting Foxo1-IL1r1 pathway (Ichiyama et al., 2016). Inactivation of miR-183/96/182 cluster in mice results in decreased production of pathogenic cytokines and decreased severity of experimental autoimmune encephalomyelitis (EAE) (Ichiyama et al., 2016). Another study suggested that miR-182 inhibits CD4^+^CD25^+^FoxP3^+^ regulatory T (Treg) cell differentiation (Wan et al., 2016); knockdown of miR-182 results in increased Tregs in peripheral lymph nodes and spleen, contributing to decreased severity of EAE in mice (Wan et al., 2016). In addition, miR-183/96/182 cluster is highly induced during B cell activation as well (Pucella et al., 2015, 2019; Li et al., 2016). In spite of its highly responsiveness to B cell activation, miR-183/96/182 ko or miR-182 ko mice have intact B-cell and T-cell development and follicular helper (Tfh) cell and germinal center (GC) B-cell population (Pucella et al., 2015, 2019; Li et al., 2016). However, miR-182 ko mice showed a delayed generation of antigen-specific antibody production at an early stage in the immunization regimen when challenged with a T-cell-dependent antigen and a complete impairment of antigen response in a T-cell-independent type II antigen challenge, suggesting that miR-182 is required for the extrafollicular antigen response (Li et al., 2016). Consistent with the functions of miR-183/96/182 cluster in immune systems, in an association study, Yu et al. (2014) reported that the pre-miR-182 nt106 A allele is associated with genetic susceptibility to Behcet’s disease and Vogt-Koyanagi-Harada (VKH) syndrome, both of which are characterized with autoimmune uveitis (Yu et al., 2014).

Collectively, these reports suggest that miR-183/96/182 cluster plays important roles in different aspects of the functions of various types of innate and adaptive immune cells and tumorigenesis, in addition to its essential roles in photoreceptors and other sensory neurons. Therefore, we predict that mutations in the miR-183/96/182 cluster and dysregulation of its expression may result in defects or phenotypic changes in different aspects of the immune system and the inflammatory/immune responses to antigen invasion and other external and internal pathological insults. Diseases caused by defects in the miR-183/96/182 are most possibly syndromic in nature with constellations of symptoms beyond either sensory or immune systems.

Considering that miRNAs are quantitative regulators of gene expression and that immune/inflammation processes play important roles in normal aging and many multi-factorial retinal diseases, e.g., autoimmune uveitis, age-related macular degeneration, diabetic retinopathy, and glaucoma (Chen et al., 2019), we predict that sequence variants and other mutations in the miR-183/96/182 cluster may contribute to pathogenesis and enhance the susceptibility to these diseases. Studies on the functional consequence of the sequence variants or other mutations of miR-183/96/182 must be conducted in a broader context including both sensory and immune system as well as neuroimmune interactions.

## Data Availability Statement

The datasets presented in this study can be found in online repositories. The names of the repository/repositories and accession number(s) can be found below: https://www.ncbi.nlm.nih.gov/snp/, rs76481776 https://www.ncbi.nlm.nih.gov/snp/, rs77586312 https://www.ncbi.nlm.nih.gov/snp/, rs80041074 https://www.ncbi.nlm.nih.gov/snp/, rs41274239 https://www.ncbi.nlm.nih.gov/snp/, and rs73159662 https://www.ncbi.nlm.nih.gov/snp/, rs73159662.

## Ethics Statement

The studies involving human participants were reviewed and approved by the IRB of Wayne State University, National Institutes of Health, and Committee for Protection of Human Subjects, University of Texas Southwestern Medical Center. The patients/participants provided their written informed consent to participate in this study.

## Author Contributions

SX: project conception, experimental design, data analysis, and manuscript preparation. AC, CM, AH, EM, CD, WL, JC, and WG: conducting the experiments and data analysis. KW and DB: sample collection and clinical data of members of Family 4100. KG: sample collection from eyeGENE. All authors contributed to the article and approved the submitted version.

## Conflict of Interest

The authors declare that the research was conducted in the absence of any commercial or financial relationships that could be construed as a potential conflict of interest.

## Footnotes

1 sph.uth.edu/Retnet

2 www.mirbase.org

3 https://eyegene.nih.gov/

4 http://www.mirbase.org/

5 http://rna.tbi.univie.ac.at/cgi-bin/RNAWebSuite/RNAfold.cgi

6 http://rna.tbi.univie.ac.at//cgi-bin/RNAWebSuite/RNAfold.cgi
