# Supplementary material for: Mutation Screening in the miR-183/96/182 Cluster in Patients With Inherited Retinal Dystrophy
Source: Front Cell Dev Biol. 2020 Dec 23;8:619641. doi: 10.3389/fcell.2020.619641 (PMC7785829; doi:10.3389/fcell.2020.619641)
Supplement: Supplementary file 1 [file Data_Sheet_1.PDF]

Supplemental material

Supplemental sequences:

1. Sequence of the 183/96F1-R1 amplicon

```
          183/96-F1                                      183/96-F2
  1  GAAGGTCATC  TTGGGCTGAT  GGGGCATGTG  GATCTTGTGA  AGAGGTGGGA  TGGGGTGGGG
     CTTCCAGTAG  AACCCGACTA  CCCCGTACAC  CTAGAACACT  TCTCCACCCT  ACCCCACCCC

 61  GGTAGAGACC  GTAGCAGCCG  CTGCTGAGGG  CCTGCTGGGG  GGCCCCCAAG  GGAGTGGGCA
     CCATCTCTGG  CATCGTCGGC  GACGACTCCC  GGACGACCCC  CCGGGGGTTC  CCTCACCCGT

121  GGCTAGGAGC  AGGGAACGGG  CATCGTGGGC  CGCTGGTCTC  TCCGCAGGGT  CGGCAGGCCG
     CCGATCCTCG  TCCCTTGCCC  GTAGCACCCG  GCGACCAGAG  AGGCGTCCCA  GCCGTCCGGC

        Pre-miR-183                    Mature miR-183
181  CAGAGTGTGA  CTCCTGTTCT  GTGTATGGCA  CTGGTAGAAT  TCACTGTGAA  CAGTCTCAGT
     GTCTCACACT  GAGGACAAGA  CACATACCGT  GACCATCTTA  AGTGACACTT  GTCAGAGTCA
                                                  Pre-miR-183
241  CAGTGAATTA  CCGAAGGGCC  ATAAACAGAG  CAGAGACAGA  TCCACGAGGG  CCTCCGGAGC
     GTCACTTAAT  GGCTTCCCGG  TATTTGTCTC  GTCTCTGTCT  AGGTGCTCCC  GGAGGCCTCG

301  ACCTTACCCA  CTTCTGCCTT  GAGTGCTCCT  AGACGTCGGA  AACAGGCTGC  TTCCAAGGGT
     TGGAATGGGT  GAAGACGGAA  CTCACGAGGA  TCTGCAGCCT  TTGTCCGACG  AAGGTTCCCA

361  GCAGGGATGC  AAGGCCCCTC  GTCCAGTGTG  TCCCCAGAGA  GCCCGCACCA  GTGCCATCTG
     CGTCCCTACG  TTCCGGGGAG  CAGGTCACAC  AGGGGTCTCT  CGGGCGTGGT  CACGGTAGAC
         Pre-miR-96        Mature miR-96
421  CTTGGCCGAT  TTTGGCACTA  GCACATTTTT  GCTTGTGTCT  CTCCGCTCTG  AGCAATCATG
     GAACCGGCTA  AAACCGTGAT  CGTGTAAAAA  CGAACACAGA  GAGGCGAGAC  TCGTTAGTAC
              Pre-miR-96
481  TGCAGTGCCA  ATATGGGAAA  AGCAGGACCC  GCAGCTGCGT  CCGCCTCCCC  TGCATCCTTG
     ACGTCACGGT  TATACCCTTT  TCGTCCTGGG  CGTCGACGCA  GGCGGAGGGG  ACGTAGGAAC

541  TGTCAGGGCC  CCAGCCTGCT  CCTCCTCAAG  GCCTCCTCAC  CGCCTCCCCA  GCCCATCTGG
     ACAGTCCCGG  GGTCGGACGA  GGAGGAGTTC  CGGAGGAGTG  GCGGAGGGGT  CGGGTAGACC

601  CTCAGCTGCT  GTGTGAGGGC  CCAGCGCTGG  TGGGCAGCCA  GATCGCCTTA  CACTGCCTGG
     GAGTCGACGA  CACACTCCCG  GGTCGCGACC  ACCCGTCGGT  CTAGCGGAAT  GTGACGGACC
                                                       183/96-R1
661  GGCCACGGTA  GAGCTGGGAG  CCCAGCAATC  TGAGCTGGGC  CAGCAGATGG  GGCCGCCCAG
     CCGGTGCCAT  CTCGACCCTC  GGGTCGTTAG  ACTCGACCCG  GTCGTCTACC  CCGGCGGGTC

721  GGCAGAGGTG  GGGGAGTCTG  AAACCATCTG  TAG
     CCGTCTCCAC  CCCTCAGAC  TTTGGTAGAC  ATC
                          183/96-R1
```

2. Sequence of the 182F0-R3 amplicon

```
              182-F0
1    TCTGGCCTGG CTTGTGCTGG TTCCCAGAGT CCCTGCTGTC AGGTGCTGGG AGTGGAGGTG
     AGACCGGACC GAACACGACC AAGGGTCTCA GGGACGACAG TCCACGACCC TCACCTCCAC

61   CCCTCGGGTT CCCTTGGGAG TGGTGACCAC AGCTCAGGGC CTGGGTTGGA TCCTGGTGGC
     GGGAGCCCAA GGGAACCCTC ACCACTGGTG TCGAGTCCCG GACCCAACCT AGGACCACCG

                                                  182-F1
121  TGCCCTAGGG ATGGTGTCTG CTCCATGCCT GCCCACAGGA ACTGCAGGTT ACAGATATGA
     ACGGGATCCC TACCACAGAC GAGGTACGGA CGGGTGTCCT TGACGTCCAA TGTCTATACT

181  GGGGAAGGGA GGAGAGGAGG GGGCTGAGGA GGGACCGGGA CCAGCAGGAA GGGGGACTGT
     CCCCTTCCCT CCTCTCCTCC CCCGACTCCT CCCTGGCCCT GGTCGTCCTT CCCCCTGACA

                                                 182-F2
241  GGGGTTGGGC CTCCACACCA GGGCGACCCT GCAGGAAGGA CCTTGTCGCA GTTGCGGGGA
     CCCCAACCCG GAGGTGTGGT CCCGCTGGGA CGTCCTTCCT GGAACAGCGT CAACGCCCCT

301  TGGGCGCCTC TGTCCTGGCC CTGCCTGGAC CATCCTAACT GTCTCTGTCT CTTCCTCAGC
     ACCCGCGGAG ACAGGACCGG GACGGACCTG GTAGGATTGA CAGAGACAGA GAAGGAGTCG
                                            Pre-miR-182
361  ACAGACCGAG GCCTCCCCAG CTCCTGGGGG GAGCTGCTTG CCTCCCCCCG TTTTTGGCAA
     TGTCTGGCTC CGGAGGGGTC GAGGACCCCC CTCGACGAAC GGAGGGGGGC AAAAACCGTT

       Mature miR-182                                Pre-miR-182
421  TGGTAGAACT CACACTGGTG AGGTAACAGG ATCCGGTGGT TCTAGACTTG CCAACTATGG
     ACCATCTTGA GTGTGACCAC TCCATTGTCC TAGGCCACCA AGATCTGAAC GGTTGATACC

481  GGCGAGGACT CAGCCGGCAC CCTGTGCACA GCCAGCGAGG GAAGGGCCGG CCATGCTGGA
     CCGCTCCTGA GTCGGCCGTG GGACACGTGT CGGTCGCTCC CTTCCCGGCC GGTACGACCT

541  CCTGCTGTTC TCCGCGAGGA AGGAGGGGAC TCAGGTCCCG GACTGCTGGG TAGTGGCAGA
     GGACGACAAG AGGCGCTCCT TCCTCCCCTG AGTCCAGGGC CTGACGACCC ATCACCGTCT

601  GGGCAGGTGC AGCTGGAAGT GACACTCTGT GTTTCCCTGC ATCCCCCTGA GGTCACAGGT
     CCCGTCCACG TCGACCTTCA CTGTGAGACA CAAAGGGACG TAGGGGGACT CCAGTGTCCA

661  CCTCAAGTCA GCTGGGAAGC C                                  182-R2
     GGAGTTCAGT CGACCCTTCG G

     182-R2     182-R3
```
